# Low-Power n-FET-Only Operational Transconductance Amplifier in Flexible IGZO TFT Technology

**DOI:** 10.3390/s26216958

**Published:** 2026-11-04

**Authors:** Juan Luis Soler-Fernández, Angel Diéguez, J. Daniel Prades, Oscar Alonso

**Affiliations:** 1 Department of Electronics and Biomedical Engineering, Universitat de Barcelona, 08028 Barcelona, Spain; 2 Institute of Semiconductor Technology (IHT), Technische Universität Braunschweig, D-38106 Braunschweig, Germany; 3 Laboratory for Emerging Nanometrology (LENA), Technische Universität Braunschweig, D-38106 Braunschweig, Germany

**Keywords:** flexible electronics, IGZO TFT, operational transconductance amplifier (OTA), ultra-low power, IoT

## Abstract

**Highlights:**

**What are the main findings?**

- An n-FET-only OTA was fabricated in a flexible 0.6 µm IGZO TFT process.
- The architecture combines resistive loading with a three-stage gain chain and does not use weak-conduction TFTs as high-side loads.
- The measured median open-loop DC gain is 56.6 dB, with a large chip-to-chip input-offset spread.
- In unity-gain feedback, the median closed-loop −3 dB bandwidth is 3.7 kHz. The average static power over the measured input range is approximately 33–53 µW at 3.3 V.

**What are the implications of the main findings?**

- The circuit demonstrates low-power analog gain in a commercial flexible IGZO process.
- The measurements also identify input offset, load-driving capability and supply coupling as relevant limitations for practical sensor interfaces.
- Environmental stress and complete energy-harvesting system operation remain to be evaluated.

**Abstract:**

This paper presents an n-FET-only operational transconductance amplifier (OTA) fabricated in a flexible 0.6 µm IGZO TFT process. The architecture combines a resistive-load differential pair with a three-stage common-source amplification chain and avoids using weak-conduction TFT loads to set the high-side currents. Open-loop measurements across 10 dies show a median DC gain of 56.6 dB and an input-offset distribution of approximately −313 ± 419 mV. The unloaded open-loop output excursion reaches 3.18 V. In a unity-gain follower configuration, the median closed-loop −3 dB bandwidth is 3.7 kHz, and stable operation is observed without an explicit compensation capacitor. The cross-chip average supply current varies from approximately 10 µA to 16 µA over the input sweep, corresponding to approximately 33–53 µW at 3.3 V. These results demonstrate a low-power IGZO amplifier for low-frequency sensor interfaces, while input offset, load-driving capability and supply coupling remain important limitations of the present implementation.

## 1. Introduction

Wearable and distributed sensor nodes on flexible substrates need analog front-ends that buffer or amplify slowly varying signals from high-impedance transducers within tight energy budgets [1,2,3]. When such nodes are powered by ambient energy harvesting (photovoltaic [4,5], thermoelectric [6,7] or piezoelectric [8,9]), the continuous power available is typically in the low-microwatt range [10,11]. For these interfaces, the amplifier properties that matter are static power, DC accuracy (offset and gain error), output drive, supply sensitivity and bandwidth for the signal of interest. Flexible integrated circuits (FlexICs) [12,13] are attractive for such nodes, and replacing rigid silicon and batteries is also motivated by the environmental impact of electronic waste [14,15]. Recent flexible a-IGZO front-ends for electrocorticography and myoelectric control illustrate this application space [16,17].

Indium Gallium Zinc Oxide (IGZO) thin-film transistors (TFTs) are a leading semiconductor technology for sustainable FlexICs [18,19,20]. Fabricated below 300 °C [21], IGZO technologies bypass high-energy thermal oxidation and doping, reducing carbon footprint, water usage, and reliance on harmful chemicals [22]. IGZO TFTs also exhibit higher electron mobility than organic semiconductors [23], ultra-low off-state leakage currents [18], and, in the same commercial process used here, characterization under static tensile stress [13] and operation of a microprocessor bent to a 3 mm radius [24].

Despite the realization of IGZO-based digital circuits [12,20,25], analog signal conditioning remains a bottleneck. Autonomous sensor nodes require analog front-ends and operational amplifiers (op-amps) to amplify and filter transducer signals [20]. However, commercial metal-oxide TFT processes lack stable p-type field-effect transistors [26,27], restricting designers to unipolar (n-FET-only) architectures. In unipolar technologies, active loads are substituted with passive resistive topologies, diode-connected n-FETs, or bootstrapping [28]. Resistive-load architectures introduce a severe trade-off: high voltage gain requires large load resistances, consuming excessive layout area and introducing thermal noise. Conversely, minimizing resistor area drastically increases static quiescent current and degrades output swing.

Recent work has also explored low-power biasing and self-biased input stages in flexible IGZO technologies. Ref. [29] compared three self-biased differential input stages using post-layout simulations, while a later fabricated three-stage OTA in the same 0.6 µm class of IGZO-on-plastic technology reported a mean DC gain of 41.2 dB, a mean gain-bandwidth product of 200 kHz and a mean power consumption of 60 µW at 3 V across six dies. These results illustrate the strong trade-off among gain, bandwidth, capacitive load, area and static power in flexible unipolar amplifiers.

Reported flexible IGZO amplifiers span a wide range of power and bandwidth. Earlier measured op-amps include milliwatt-level implementations, while more recent circuits have reached sub-100-µW operation under specific supply and load conditions. These results motivate a design focused on low static power and low-frequency operation, while also showing that power must be compared together with gain, bandwidth, load capacitance, area and measurement conditions [30,31]. These static continuous current draws exceed the generation capacity of sustainable micro-harvesters, rendering current TFT op-amps incompatible with eco-friendly IoT nodes. Furthermore, analog circuits on unpassivated flexible substrates suffer from process variability, threshold voltage shifts under thermal stress, and photosensitive leakage currents [20,32,33].

This paper reports the design and multi-die electrical characterization of a resistively loaded n-FET-only OTA fabricated in the PragmatIC 0.6 µm IGZO process. The circuit uses an established topology of a resistively loaded differential pair, a source-follower level shifter and three common-source stages, designed for operation in the tens-of-microwatts range at 3.3 V. The contribution is not a new topology but a quantitative account of what limits such an amplifier in practice. Open-loop and closed-loop DC behavior, load dependence, supply current, frequency and step response, and supply coupling were measured on up to ten identified dies, and the results are linked to a first-order analysis of the circuit. The analysis identifies where the static power is spent, why the input-referred offset is large, and what sets the speed and stability of the follower.

The remainder of this paper is organized as follows. Section 2 details the circuit design methodology and the sizing strategies required to overcome unipolar technology constraints. Section 3 presents the experimental measurement setup, the measured sample-to-sample dispersion, and the conditions that were not tested (validation boundary). Section 4 provides a discussion of these results, benchmarking the proposed design against existing flexible analog interfaces. Finally, Section 5 concludes the manuscript.

## 2. Circuit Design Methodology

The proposed OTA provides stable, low-power analog signal conditioning for flexible electronic systems. Fabricated in a 0.6 µm IGZO TFT process from PragmatIC, the design is constrained to unipolar n-FET devices. Figure 1 (top) illustrates the complete transistor-level schematic. The architecture is partitioned into three functional blocks: a resistive-load differential input pair, a symmetric source-follower level shifter, and a three-stage common-source amplification chain. The design methodology prioritizes predictability and ultra-low static power over architectural complexity to meet the strict energy budgets of micro-harvester-powered IoT nodes.

In complementary CMOS technologies, high voltage gain and low static power are achieved using p-type active current mirrors. Lacking p-FETs, unipolar technologies force a choice between passive resistive loads and n-FET-based active loads. Alternative high-impedance unipolar implementations frequently replace passive resistors with diode-connected TFTs or transistors biased in the cut-off regime to minimize layout area and static current [28,34]. In the same process, a leakage-based timer showed a large chip-to-chip spread (mean wake-up frequency 0.24 ± 0.15 Hz across 13 chips) [13], which indicates that leakage-defined operating points vary strongly between dies. Loads biased near cut-off would therefore set operating points that are difficult to predict. Diode-connected loads, conversely, restrict the available output voltage swing due to their mandatory threshold voltage drop and intrinsically limit the stage voltage gain.

To circumvent these limitations, this OTA utilizes high-value passive resistors (R0–R5) for all biasing and load networks. This choice increases the footprint, but it makes the tail current of the input pair and the load currents of the gain stages resistor-defined and avoids the threshold drop of diode-connected loads. It does not make the whole bias network resistor-defined: as quantified below, most of the static current flows in the source-follower level shifter.

The input stage consists of a source-coupled differential pair (M3, M5) biased by a current mirror (M0, M4) and a reference resistor (R0). Passive resistors R1 and R2 serve as the loads for the differential branches.

Following the input stage, the differential signals must be converted to a single-ended output while simultaneously shifting the DC bias level down to keep the subsequent amplification stages in saturation. This is achieved through a combined level-shifter and active load structure (M1, M2, M6, M7). The left differential branch drives a source follower (M1) in series with a current mirror (M2, M7). Simultaneously, the right differential branch drives the source follower M6. Because M7 acts as the active current sink for M6, the differential signals sum constructively at the output node of M6–M7. This topology efficiently performs the differential-to-single-ended conversion while preserving the signal gain.

The reference current,

(1) $$I_{\mathrm{REF}}=\frac{\mathrm{VDD}-V_{\mathrm{GS}0}}{R_{0}}$$

is copied 1:1 by M4 into the tail of the input pair, so that each input branch carries *I*_3,5_ = *I_REF_*/2 and each load resistor drops *R*_1_ · *I*_3,5_. The common-source stages draw at most *VDD*/*R_k_* = 660 nA each. At *VDD* = 3.3 V the resistor branches can therefore carry at most 2.3 μA in total. The level shifter is different: M1–M2 and M6–M7 are connected between *VDD* and ground, and their common current *I_LS_* is set by the stacked gate-source voltages

(2) *V_GS_*_1_ + *V_GS_*_2_ = *V_dL_* ≈ *VDD* − *R*_1_ · *I*_3,5_.

*I_LS_* therefore depends on the threshold voltage and mobility of the TFTs rather than on a resistor. Comparing this bound with the measured supply current (Section 3.3) shows that 63–90% of the static current flows in the level shifter, which also explains the die-to-die spread of the supply current.

Neglecting output conductances, the source follower M1 loaded by the diode-connected M2 gives

(3) $$V_{b}=\frac{g_{m1}}{g_{m1}+g_{m2}}\cdot V_{\mathrm{dL}},$$

and the node *z* at the M6–M7 junction gives

(4) $$V_{z}\approx V_{\mathrm{dR}}-\left(\frac{g_{m7}}{g_{m6}}\right)\cdot V_{b}.$$

With M6 matched to M1 and M7 matched to M2, the gain from the differential input to *z* is

(5) $$A_{z}\approx \left[1+\frac{g_{m2}}{g_{m1}+g_{m2}}\right]\cdot \frac{g_{m3}R_{1}}{2}.$$

Each of the three common-source stages adds

(6) *A_k_* ≈ −*g_mk_*(*R_k_*||*r_ok_*).

Because the first-stage loads drop only 0.3 V,

(7) $$g_{m3}R_{1}=\left(\frac{g_{m3}}{I_{3}}\right)\cdot \left(I_{3}R_{!}\right)$$

is only a few *V*/*V* for TFT *g_m_*/*I*. This low gain matters for the DC operating point. At balance *V_dL_* = *V_dR_* and, with *M*6 = *M*1 and *I*_6_ = *I*_7_ = *I*_2_, node *z* settles at *V_z_*_0_ = *V_GS_*_2_(*I_LS_*). The first common-source stage, however, requires V_z__*_ = *V_GS_*_8_(*I*_8_) to place the output at mid-supply, with

(8) $$I_{8}=\frac{\mathrm{VDD}-V_{\mathrm{GS}9}}{R_{3}}.$$

The two voltages are set by different devices biased by unrelated mechanisms, so their difference is not removed by layout matching. It appears at the input as a systematic offset ≈(*V_z_*_0_ − *V*_z*_)/*A_z_*, which varies with the global threshold voltage and mobility of each die. Random mismatch of the input pair and of the load resistors adds to this term.

Because this tape-out did not include isolated test transistors, single-device parameters could not be extracted directly from the characterized dies. To provide context for this operating-point analysis, the measured transfer and output characteristics of isolated TFTs fabricated in this commercial process are detailed in [13]. These prior empirical measurements support the nominal transconductance and bias voltages used in the first-order analysis above, though they do not capture the local device-to-device mismatch present within the fabricated OTA itself.

However, unipolar IGZO TFTs possess relatively low intrinsic transconductance compared to bulk silicon MOSFETs. Consequently, a single amplification stage is insufficient to achieve the open-loop gain mandated for accurate closed-loop sensor signal conditioning. To overcome this, the output of the level shifter cascades into a three-stage common-source chain (M8–M10 and R3–R5). Each successive stage acts as an inverting amplifier, multiplying the overall open-loop gain to recover the loss from the single-ended conversion and maximizing the available output voltage swing.

The sizing of this final chain defines the fundamental power-bandwidth trade-off of the OTA. Achieving high voltage gain in a resistive-load common-source stage requires maximizing the load resistance. The resistive loads (R3–R5) and the channel widths of M8–M10 were iteratively sized to minimize the total quiescent current drawn from the supply.

No compensation capacitor is used. The internal high-impedance nodes (the drains of M3, M5, M8 and M9) are loaded by 5 MΩ resistors and only device and interconnect capacitance, whereas the output node (*R*5||*r_o_*_10_ ≈ 5 MΩ) also carries the capacitance of whatever the amplifier drives. In the measurements reported here, that capacitance is dominated by the test setup (≈0.4 nF) which places the dominant pole at

(9) $$f_{p}=\frac{1}{2\pi R_{5}C_{L}}$$

below the internal poles. While this restricts the unity-gain bandwidth to the low-kilohertz regime (sufficient for quasi-static IoT sensing), it supports stable closed-loop operation while preserving significant layout area.

Figure 1 (bottom left) presents the physical layout of the OTA alongside a micrograph of the fabricated die (Figure 1 (bottom right)). The core amplifier occupies an active area of 400 μm × 320 μm, excluding the peripheral bond pads required for direct probing. To mitigate the severe process variations and threshold voltage mismatch inherent to unpassivated flexible substrates, strict physical symmetry was enforced during the layout of the differential input pair and the symmetric source-follower branches. Components were placed in close spatial proximity with matched routing to minimize systematic offsets and ensure identical thermal and mechanical stress profiles across the differential paths. Furthermore, the high-value passive resistors required for the unipolar architecture dominate the total area. The resistors were implemented using a dedicated 200 kΩ/sq resistor layer with a minimum line width and spacing of 1.4 μm. According to the process extraction, this meandering introduces an estimated parasitic capacitance of 126 fF. The tolerance in this kind of technology is high [35] and, for this specific process, the foundry specifies a nominal sheet resistance tolerance of 40% and a linear temperature coefficient of −0.6% per °C for this layer. The clipping level gives an in situ estimate of R5 for each die: *R*_5_ = [5.01 ± 0.42] MΩ. Layout symmetry addresses matched pairs only. The DC interface between the level shifter and the first common-source stage is not ratiometric (see the offset analysis above), so global variations in threshold voltage and mobility also contribute to the input-referred offset. Because single-device characteristics could not be measured on these chips, the available circuit-level data do not independently separate the individual contributions of local threshold voltage mismatch, global mobility variation, or time-dependent charge-trapping effects.

## 3. Experimental Characterization

This section details the electrical characterization of the fabricated IGZO OTA. Up to 10 physical dies were characterized. The number of available samples differs between measurement types and is reported for each result. The evaluation covers open-loop DC transfer characteristics, closed-loop DC and transient behaviors, AC frequency response, and power-supply rejection ratio (PSRR).

### 3.1. Experimental Setup

The physical dies were contacted and tested on a vibration-isolated needle station. Direct probing of the bond pads was utilized to ensure the most reliable electrical access to the unencapsulated flexible circuits. Figure 2a shows the complete experimental setup, which was fully automated using a control PC running custom Python (3.10) scripts to control the instrumentation. All measurements were performed at room temperature (25 °C) in dark conditions, on unpackaged dies in the flat state.

The closed-loop measurements were performed without numerical offset correction. The same die identifiers were retained across the open-loop and closed-loop datasets where measurements were available, allowing the measured offset and follower response to be compared at sample level.

An Agilent N6705 Power Analyzer (Agilent Technologies Inc., Santa Clara, CA, USA). and an Agilent B2912A Source/Measure Unit (SMU) (Agilent Technologies Inc., Santa Clara, CA, USA). provided the supply rails and measured the static current consumption. Dynamic input signals were driven by an Agilent 33250A function generator (Agilent Technologies Inc., Santa Clara, CA, USA)., and a Keysight MSOX6004A oscilloscope (Agilent Technologies Inc., Santa Rosa, CA, USA). captured the resulting output waveforms. To prevent the input impedance of the test equipment from loading the OTA and distorting the measured signals, a commercial high-input-impedance amplifier (OPA2182, Texas Instruments, Dallas, TX, USA) was configured as a voltage follower (supplied at 6 V) and placed in series between the OTA output pad and the oscilloscope.

The OPA2182 is used only as a measurement buffer. Because its unity-gain small-signal bandwidth is approximately 5 MHz, quantitative frequency-domain conclusions are restricted to the range in which the buffer and oscilloscope chain do not significantly affect the measured transfer (i.e., 1 MHz).

To extract the electrical characteristics of the OTA, the Device Under Test (DUT), three distinct measurement configurations were implemented. For the open-loop DC characterization (Figure 2b), the inverting terminal was held at a constant 1.65 V mid-supply reference while the non-inverting terminal was swept across the 0 V to 3.3 V range. To evaluate closed-loop DC, small-signal AC frequency response, and large-signal transient behavior, the OTA was configured as a unity-gain voltage follower, with the output tied directly to the inverting input (Figure 2c). Finally, for the Power-Supply Rejection Ratio (PSRR) measurements, the amplifier was maintained in the unity-gain configuration while a frequency-swept 50 mV_pp_ AC ripple was superimposed onto the 3.3 V DC supply rail to quantify the coupled output variation (Figure 2d).

### 3.2. Open-Loop DC Response

To evaluate the intrinsic gain and input offset voltage (*V_OS_*) of the amplifier, the OTA was configured in an open-loop measurement setup. Figure 2b illustrates this configuration, where the inverting terminal was biased at a constant reference voltage of 1.65 V (half-supply) while a DC sweep from 0 V to 3.3 V was applied to the non-inverting terminal.

Figure 3a presents the measured output voltage (*V_OUT_*) against the input voltage ($V_{IN_{P}}$) for 10 fabricated dies under a no-load condition. The output exhibits a sharp transition from the lower to the upper supply rail, confirming the high intrinsic open-loop gain provided by the three-stage common-source amplification chain.

However, the input voltage threshold at which this transition occurs varies significantly across the samples. The inset histogram in Figure 3a quantifies this input offset voltage, revealing a mean offset (μ) of −312.61 mV and a standard deviation (σ) of 418.7 mV. The ten values range from [−1.05] to [+0.18] V (median −0.21 V). With *n* = 10, the 95% confidence interval of σ is 0.29–0.76 V. The non-zero mean indicates a systematic component in addition to random mismatch; Section 2 relates it to the non-ratiometric interface between the level shifter and the first gain stage.

Figure 4a extracts the maximum DC open-loop gain for each individual chip. The measured gain ranges from 54.0 dB to 59.6 dB, with a median value of 56.6 dB. The gain remains within 54.0–59.6 dB across the measured open-loop samples, while the much larger spread in input offset remains an important limitation of the present implementation.

### 3.3. Closed-Loop DC Response and Power Consumption

To evaluate closed-loop linearity and output swing, the OTA was configured as a unity-gain voltage follower (Figure 2c). Figure 3b presents the DC transfer characteristics for the fabricated dies under a no-load condition. The output tracks the input linearly across the mid-supply region. Signal clipping occurs near the ground rail, fundamentally limited by the threshold voltage of the NMOS differential input pair, and near the positive supply rail due to the voltage drop across the output stage resistive loads.

Figure 3c demonstrates the impact of external loading on the available output voltage swing. The DC response was measured across different load conditions, ranging from −1000 nA (sourced by the OTA) to +1000 nA (sunk by the OTA). Because the only pull-up path at the output is R5, the OTA can source at most (*VDD* − *V_OUT_*)/*R*_5_ ≈ 0.33 μA at mid-supply, while sinking currents are absorbed by M10. With a −100 nA load, the output therefore clips at *VDD* − 0.1 μA · *R*_5_ ≈ 2.8 V (Figure 3c), and with −1000 nA it cannot be held near mid-supply (Figure 4b).

This load dependency is further quantified in Figure 4b, which illustrates the output voltage deviation (Δ*V_OUT_*) at a constant mid-supply input (*V_INp_* = 1.65 V) across the evaluated load currents. Figure 4b shows the deviation at V_INP_ = 1.65 V. The same-sign deviations observed for opposite load polarities suggest that sweep-to-sweep variation may contribute alongside load dependence. The available measurements do not independently separate these contributions. At +1000 nA, the magnitude of the average deviation is approximately 90 mV, while at −1000 nA, the output collapses, as expected from the 0.33 μA sourcing limit.

The total supply current (*I_DD_*) was recorded during the DC sweep. Figure 3d shows that the cross-chip average decreases from approximately 16 µA to 10 µA as the input voltage increases. At 3.3 V, this corresponds to an average static power of 53 µW to 33 µW over the measured input range. The decrease with V_IN_ is consistent with the input common-mode level: as the tail current increases, the drop across R1 and R2 grows and the gate drive of the level shifter falls. The follower transfer characterizes closed-loop DC accuracy; CMRR and input-referred noise were not independently characterized due to limitations in the characterization setup.

### 3.4. Voltage Follower Transient Response

The large-signal dynamic behavior of the OTA was evaluated by applying a 3.3 V step input to the unity-gain voltage follower configuration. Figure 5 presents the measured time-domain step response across the sample dies.

The output exhibits a highly asymmetric dynamic response, recording an average rising slew rate *SR_rise_* = 0.69 ± 0.09 V/ms and a falling rate *SR_fall_* = 75.7 ± 2.8 V/ms. The asymmetry follows from the output stage. For a rising step, the loop saturates, M10 turns off and the output node charges only through R5. The rising edge is accordingly an RC exponential rather than a constant slew: a fit to the cross-chip mean gives *τ* ≈ 2 ms, i.e., a total output capacitance *C_OUT_* ≈ 0.4 nF. This is two orders of magnitude above the on-chip parasitics and therefore dominated by the source-measure unit and cabling connected to the output. The quoted *S_Rise_* is the 10–90% average of this exponential. For a falling step, M10 is driven fully on and discharges the same capacitance with 30 μA. At mid-supply, the largest current R5 can deliver is (*VDD* − *V_OUT_*)/*R*_5_ ≈ 0.33 μA, which also limits the sourcing load current and the slope of rising small signals. Furthermore, consistent with the DC characterization, the steady-state output does not reach the full 3.3 V upper rail due to the inherent voltage drop across the output stage resistance.

### 3.5. Voltage Follower AC Response

The small-signal frequency response of the OTA was evaluated in a unity-gain closed-loop configuration. Figure 6a presents the measured Bode magnitude plots across the fabricated dies. The closed-loop gain maintains a 0 dB baseline at low frequencies before exhibiting a −20 dB/decade roll-off. Figure 6b quantifies the closed-loop −3 dB bandwidth for each sample, which ranges from 3.1 kHz to 4.6 kHz with a median of 3.7 kHz.

The response also reveals a closed-loop gain peaking *M_P_* = 0.87 ± 0.24 dB near the cutoff frequency. This peaking provides an independent, frequency-domain method to evaluate the closed-loop stability. To extract the phase margin, the measured peaking in decibels is first converted to a linear magnitude scale

(10) $$M_{P,\mathrm{lin}}=10^{\frac{M_{P}}{20}}.$$

Using second-order control theory [36], the damping ratio (*ζ*) is calculated as

(11) $$\zeta =\sqrt{\frac{1-\sqrt{1-M_{P,\mathrm{lin}}^{-2}}}{2}}.$$

The phase margin is calculated from the damping ratio [37]

(12) $$\mathrm{PM}=\arctan\left(\frac{2\zeta }{\sqrt{\sqrt{4\zeta^{4}+1}-2\zeta^{2}}}\right).$$

Applying these equations to the average measured peaking of 0.87 dB results in a calculated phase margin of 54.6°.

The closed-loop gain peaking can also be fitted with a second-order response, giving an estimated phase margin of approximately 54.6°. This estimate is a model-based indicator rather than direct loop-gain measurements. The result established directly by the AC experiment is stable unity-gain follower operation with a median closed-loop −3 dB bandwidth of 3.7 kHz under the tested conditions.

### 3.6. Power Supply Rejection Ratio (PSRR)

The immunity of the OTA to supply variations was evaluated by superimposing a 50 mV_pp_ AC ripple on the 3.3 V supply and measuring the resulting output variation. The power-supply rejection ratio (PSRR) was calculated as

(13) $$\mathrm{PSRR}=20\cdot \log\left(\frac{V_{\mathrm{DD},\mathrm{ac}}}{V_{\mathrm{out},\mathrm{ac}}}\right),$$

such that larger positive values indicate stronger rejection. Figure 7a presents the resulting frequency response across 6 characterized dies.

At 10 Hz, the current data correspond to a PSRR of −3.45 dB. This indicates that low-frequency supply ripple is slightly amplified at the output. The rejection improves with frequency, reaching approximately 45–55 dB between 10 kHz and 1 MHz.

This susceptibility is a fundamental consequence of the n-FET-only architectural constraints. In standard complementary CMOS, active p-type current mirrors and loads provide high small-signal impedance, isolating the signal path from the supply rail. Lacking p-FETs, this IGZO design relies on passive resistors tied directly to the power supply. Specifically, the supply rail directly feeds:

• The resistor and diode-connected NMOS network that biases the tail current of the input differential pair;
• The passive resistive loads of the differential pair;
• The high-side resistive loads of all three common-source amplification stages.

Because the entire biasing and load network is resistively connected to the supply, any DC or low-frequency fluctuation on the rail immediately shifts the bias points of every stage, modulating the currents and coupling directly into the signal path.

As the frequency of the supply ripple increases, the PSRR transitions to positive values, indicating increasing rejection. Between approximately 10 kHz and 1 MHz, the measured rejection reaches about 45–55 dB. In this mid-band region, intrinsic parasitic capacitances shunt the coupled AC supply noise to ground, effectively lowering the impedance of the internal nodes and restoring noise rejection.

### 3.7. Environmental Validation Boundary

All results were obtained on unpackaged dies, directly probed in the flat state, at room temperature (25 °C) in dark conditions. The following conditions were not tested on this OTA: mechanical bending (static or cyclic), temperature, illumination changes, prolonged bias stress and long-term operation. For another circuit in the same process, static tensile stress (convex radii of 10 and 5 mm), temperature (15–45 °C) and illumination were characterized in [13]; illumination changed the output of that leakage-based timer by a factor of two, with a settling time above one hour. These results are cited only to identify which stresses should be evaluated for the present OTA; they are not used to infer its gain, offset, bandwidth, power or supply rejection under those conditions.

## 4. Discussion

To contextualize the performance of the proposed unipolar OTA, Table 1 compares the present circuit with measured flexible a-IGZO amplifiers reported in the literature. Because the reported circuits use different amplifier classes, loads, supplies and frequency metrics, the values are compared using the definitions given in the original publications.

The table should not be used to claim that the present circuit has the highest gain or the best overall performance. Ref. [31] reports 57 dB open-loop gain, while the recent [29] OTA demonstrates 60 µW mean power at 3 V with substantially higher bandwidth and a quantified 40 pF load. The present circuit instead occupies a different point in the trade-off space, combining a median 56.6 dB open-loop DC gain with tens-of-microwatts static power and low-kilohertz closed-loop operation. The comparison also shows why frequency definitions must be kept explicit: the 3.7 kHz value in this work is a closed-loop −3 dB bandwidth, whereas several literature values are open-loop unity-gain frequencies or gain-bandwidth products.

Traditional flexible operational amplifiers frequently consume power in the milliwatt regime [30] or hundreds of microwatts [38,39] to achieve wide bandwidths and stability. Even when utilizing comparable 0.6 µm a-IGZO TFT nodes, complex gain-boosted topologies report power consumption of 320 µW [38]. For the present circuit, the measured cross-chip average static power spans between 33 μW and 53 µW over the input sweep at 3.3 V. Direct power comparisons must also account for the different supply voltages, loads and amplifier architectures used in the literature. Despite this strict power budget, the OTA achieves a median open-loop DC gain of 56.6 dB while operating in the tens-of-microwatts power range.

Furthermore, unipolar resistive-load architectures typically suffer from severely degraded output voltage swings. The unloaded open-loop transfer spans 3.18 V. This value characterizes the large-signal output excursion of the open-loop sweep and should not be interpreted as the usable linear closed-loop output range. The follower measurements in Figure 3b,c provide the appropriate characterization of the output range under load.

The measured unity-gain follower has a median closed-loop −3 dB bandwidth of 3.7 kHz. This value is lower than the open-loop unity-gain frequencies or gain-bandwidth products reported by several comparison circuits [30,39], but those metrics are not directly equivalent. To account for differences in capacitive loading, a current-normalized small-signal figure of merit

(14) $$\mathrm{Fo}M_{S}=\frac{\mathrm{GBW}\cdot C_{L}}{I_{\mathrm{DD}}}$$

is considered for this work and [29]. For the present OTA, using the measured closed-loop −3 dB bandwidth as an estimate of GBW, the approximately 0.4 nF load inferred from the transient response, and the reported supply-current range gives an estimated FoM_S_ of 90–150 MHz·pF/mA, compared with approximately 400 MHz·pF/mA for [29]. This comparison suggests that part of the large difference in absolute bandwidth is associated with the different loading conditions. However, the estimated load and the use of closed-loop bandwidth instead of directly measured GBW introduce uncertainty. The result does not establish operation or stability with smaller loads, which were not tested.

Diode-connected n-FET loads limit swing and gain, and loads biased near cut-off make the operating point depend on leakage, which varies strongly between dies in this process [13]. Resistive loads avoid both problems at the cost of area, slow pull-up and direct supply coupling. The measurements show, however, that resistive loads alone do not make the bias process-independent: most of the static current, and its 2–3× die-to-die spread, comes from the V_GS_-stacked level shifter. A resistor- or mirror-defined current for the level shifter would therefore be expected to reduce both the power and its spread, and a ratiometric DC interface between the level shifter and the first gain stage, or a higher first-stage gain, would reduce the systematic part of the offset. For practical sensor interfaces, this offset necessitates either AC coupling or the integration of autozeroing techniques [16].

The results demonstrate that n-FET-only flexible IGZO technology can support low-power analog amplification with useful DC gain for low-frequency sensor interfaces. The measured offset, load-driving capability and supply coupling remain important design constraints. Integration into a battery-free or energy-harvesting node requires evaluation at complete-system level.

The dies characterized here were unpackaged and accessed by direct probing. Chips from the same platform have been connected to flexible PCBs with anisotropic conductive epoxy [16], and a microprocessor from the same platform has operated on a flexible PCB while bent to a 3 mm radius [24]. A packaged, mechanically stressed qualification of the present OTA remains to be done.

## 5. Conclusions

This work presented the design and multi-die electrical characterization of an n-FET-only operational transconductance amplifier fabricated in a 0.6 µm IGZO TFT technology on a flexible substrate and measured in the flat state. The architecture uses a resistively loaded differential pair, a source-follower level shifter and a three-stage common-source chain.

At a 3.3 V supply, the measured cross-chip average static power varies from approximately 33 µW to 53 µW over the input sweep. Despite this strict power budget, the amplifier achieves a median open-loop DC gain of 56.6 dB and an unloaded open-loop output swing of 3.18 V. Stable unity-gain follower operation was observed without an explicit compensation capacitor. The measured median closed-loop −3 dB bandwidth is 3.7 kHz, while second-order estimates derived from gain peaking give phase-margin indicators of approximately 54.6°.

The resistive loads avoid leakage-defined operating points but cause the asymmetric step response, the limited sourcing current (≤0.33 µA at mid-supply) and the direct supply coupling. Most of the static current and its die-to-die spread originate in the V_GS_-stacked level shifter, and the input-referred offset contains a systematic component from the non-ratiometric interface between the level shifter and the first gain stage. A resistor- or mirror-defined level-shifter current and a ratiometric DC interface are therefore the main design changes suggested by this study. The measurements demonstrate a low-power IGZO amplifier for low-frequency operation. The observed input-offset variation, limited load-driving capability and supply coupling define the main constraints for its use in sensor interfaces. Further characterization is required to establish its performance under environmental stress, prolonged operation and complete energy-harvesting system conditions.

## Figures and Tables

**Figure 1:**
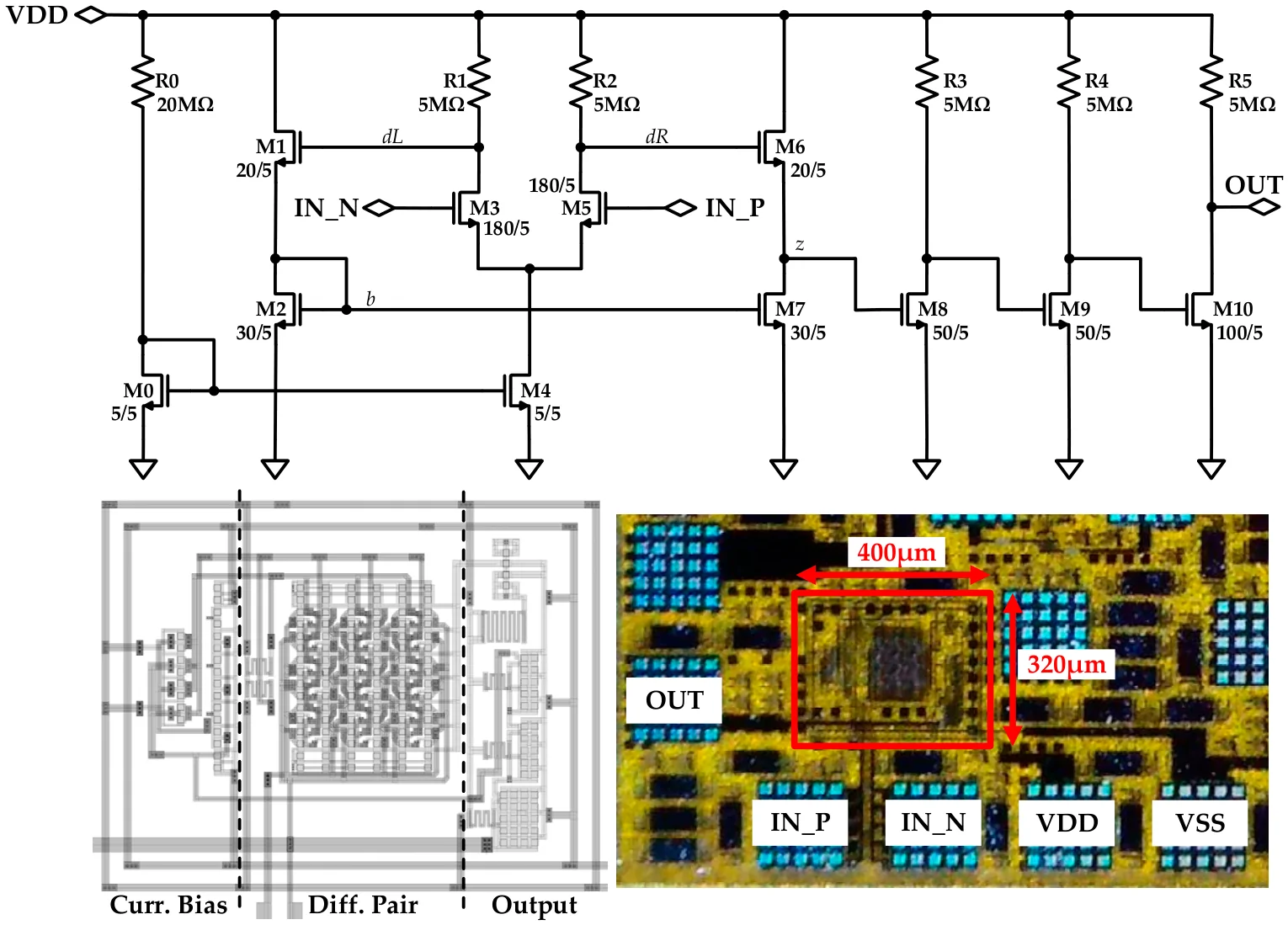
Implementation of the proposed OTA. (**Top**) Transistor-level schematic detailing the device dimensions W/L (in µm) and passive resistive load values. (**Bottom left**) Physical layout of the circuit, illustrating the separation of the current bias, differential pair, and output stage blocks. (**Bottom right**) Micro-photograph of the fabricated die highlighting the OTA core in red and the respective electrical probing pads. The active core occupies 400 × 320 µm (0.128 mm^2^), excluding the peripheral probing pads.

**Figure 2:**
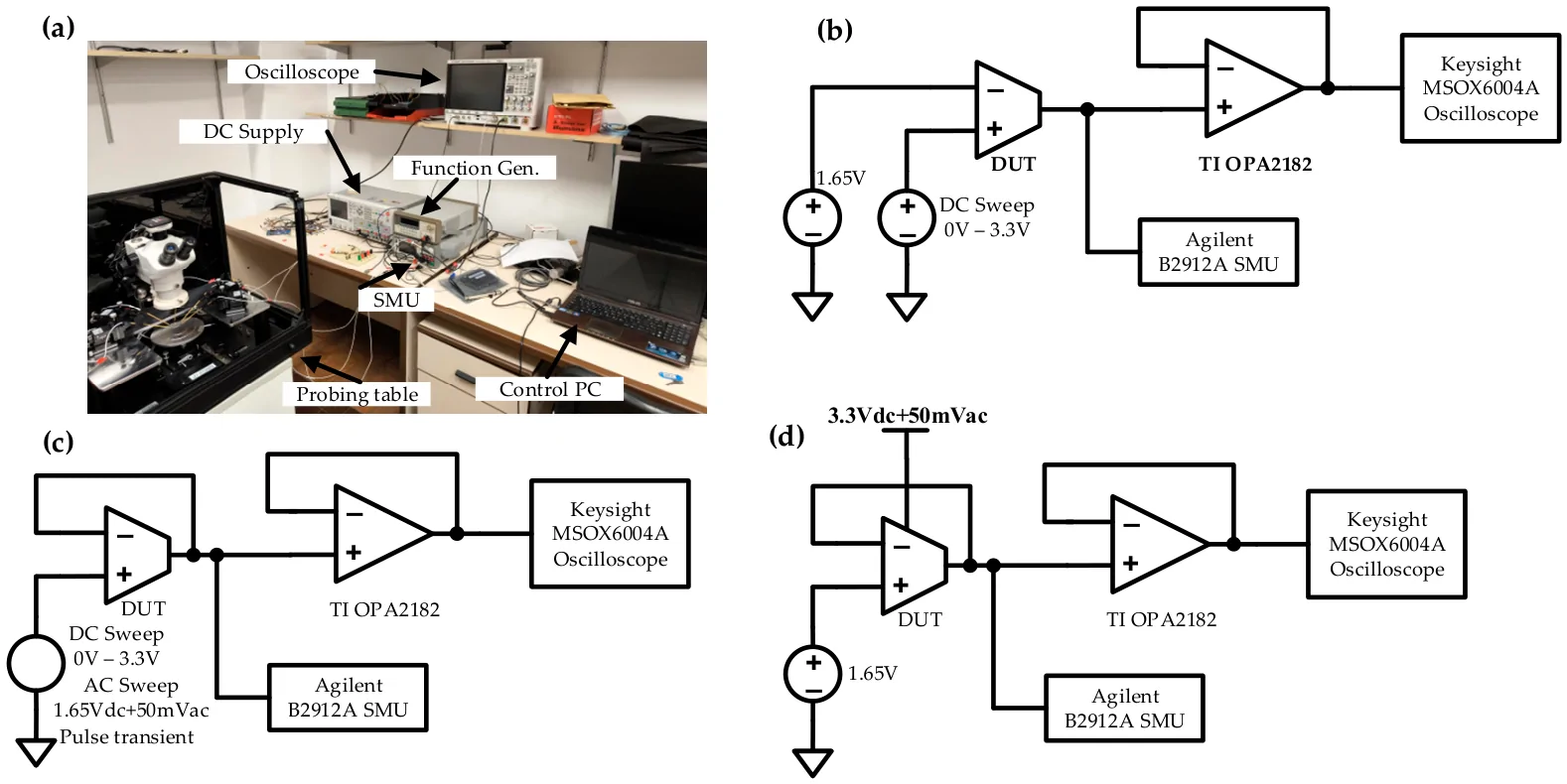
Experimental setup and measurement configurations for the characterization of the OTA. (**a**) Photograph of the automated laboratory testbench, including the vibration-isolated needle station, control PC, and measurement instrumentation. (**b**) Schematic of the open-loop DC characterization setup. (**c**) Schematic of the unity-gain voltage follower configuration utilized for closed-loop DC, small-signal AC, and large-signal transient measurements. (**d**) Schematic of the power-supply rejection ratio (PSRR) measurement setup. In all test configurations, a high-input-impedance buffer isolates the device under test (DUT) to prevent loading effects from the oscilloscope.

**Figure 3:**
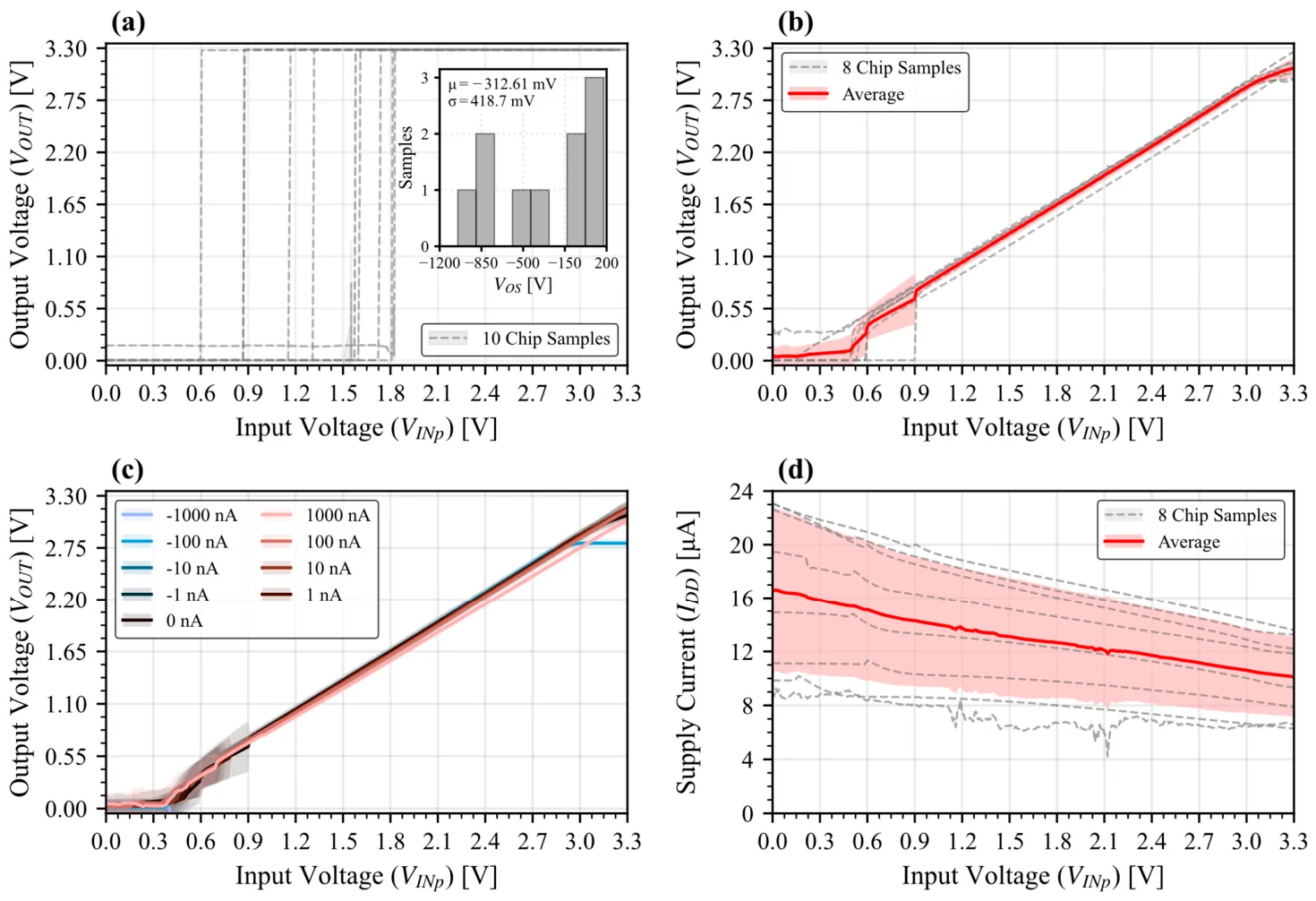
Measured DC large-signal characteristics of the fabricated OTA across multiple chip samples. (**a**) Open-loop transfer function with $V_{IN_{N}}=1.65\;V$, the inset displays the measured input offset voltage (*V_OS_*) mismatch distribution. (**b**) Closed-loop unity-gain buffer response under a zero-load condition (*I_LOAD_* = 0 nA). (**c**) Closed-loop response across a sweep of sinking and sourcing load currents ranging from −1000 nA to 1000 nA. (**d**) Static supply current consumption I_DD_ versus input voltage for the zero-load state. Where applicable, gray traces represent individual chip measurements, while solid red lines and shaded regions denote the cross-chip mean and ±1σ standard deviation.

**Figure 4:**
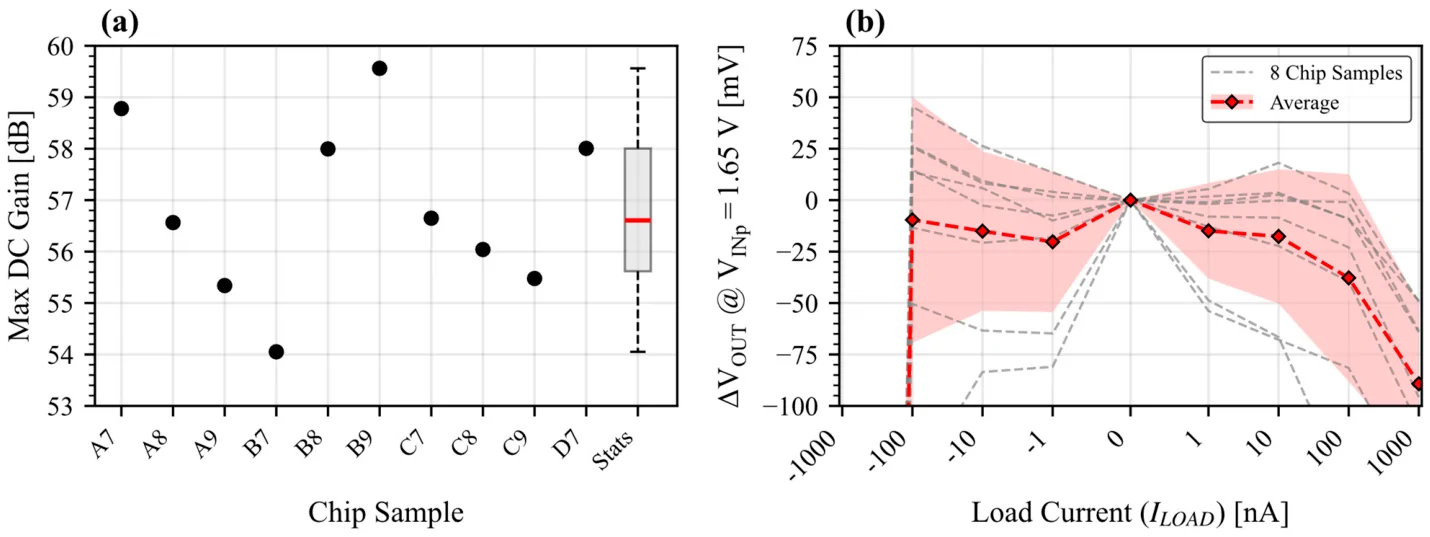
Extracted DC performance metrics and statistical variations across the measured batch. (**a**) Maximum small-signal DC open-loop gain. Discrete points represent individual peak gains, while the box plot summarizes the median, interquartile range, and outlier bounds. (**b**) Load regulation error Δ*V_OUT_* evaluated at a mid-rail input bias $V_{IN_{P}}=1.65\;V$ across the full range of applied load currents. The cross-chip average is indicated by the solid red line with a ±1σ shaded error band.

**Figure 5:**
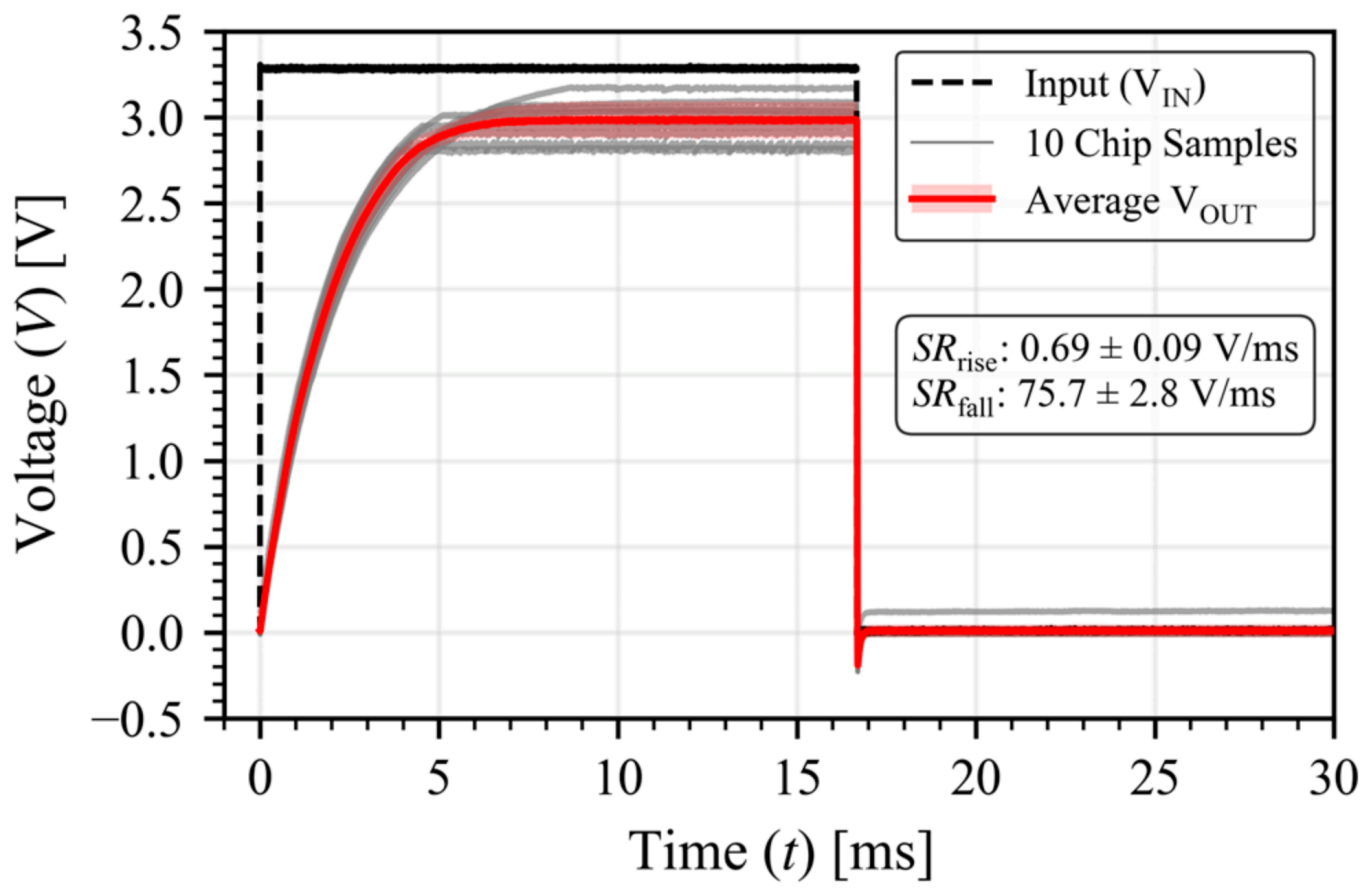
Measured transient response and dynamic performance in a unity-gain voltage follower configuration. Time-domain step response to a square-wave input pulse (*V_IN_*). Gray traces show the measured output voltage (*V_OUT_*) of individual chip samples (*n* = 10). The cross-chip mean is shown as a solid red line with a ±1σ shaded error band.

**Figure 6:**
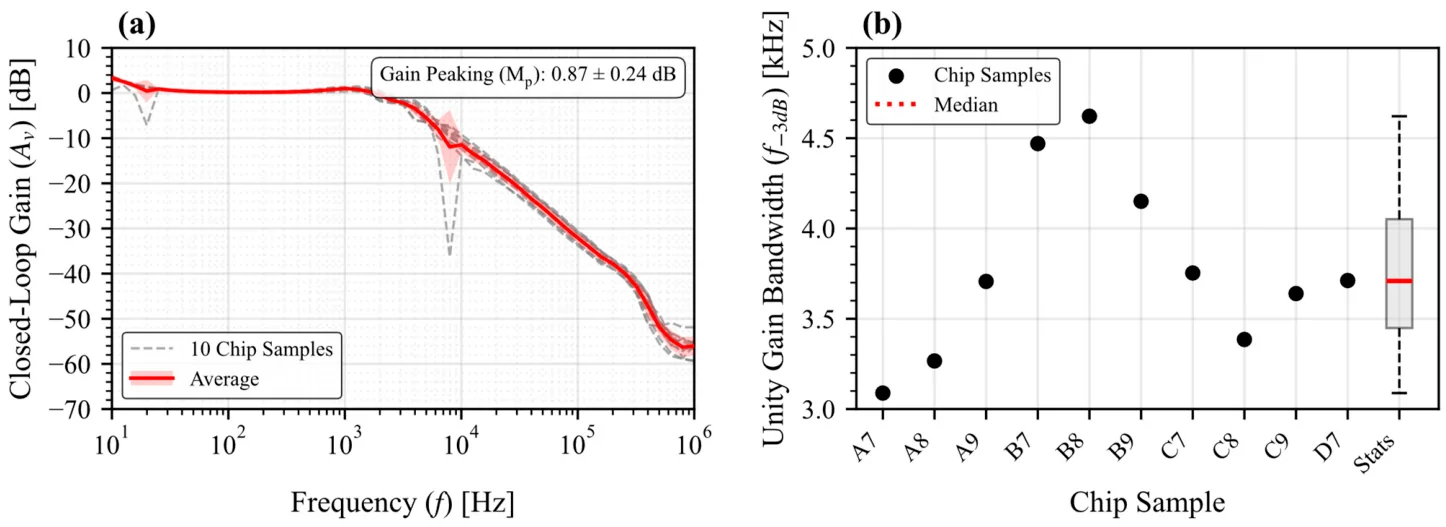
Measured closed-loop AC response of the OTA configured as a unity-gain buffer. (**a**) Gain plot. Gray traces indicate individual chip measurements (*n* = 10), while the solid red line and shaded region denote the cross-chip mean and ±1σ standard deviation. (**b**) Extracted closed-loop −3 dB bandwidth in the unity-gain follower configuration. The statistical distribution is summarized by the box plot, showing the median (solid red line) and interquartile ranges (gray box) across the fabricated batch.

**Figure 7:**
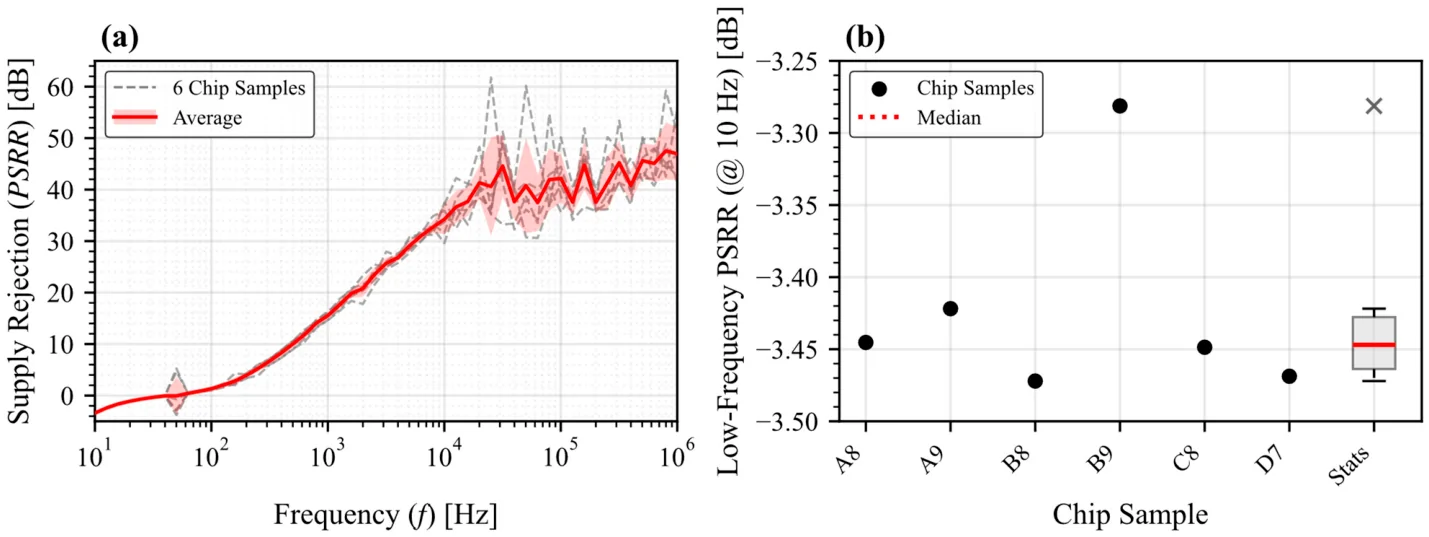
Measured (PSRR). (**a**) PSRR frequency response from 10 Hz to 1 MHz. Gray traces indicate the sweeps of individual chip samples (*n* = 6), while the cross-chip mean and ±1σ standard deviation are represented by the solid red line and shaded region, respectively. (**b**) Low-frequency PSRR extracted at 10 Hz across the measured batch. Discrete points represent individual measurements, and the box plot outlines the median (solid red line), interquartile range (gray box), and outlier boundaries (× points).

**Table 1:** Performance comparison of the proposed OTA with state-of-the-art operational amplifiers fabricated on flexible a-IGZO TFT technologies.

Parameter	This Work	[29]	[38]	[39]	[31]	[30]	[40]
Circuit/Substrate	n-FET-only OTA; PragmatIC 0.6 µm IGZO; flexible plastic	Self-biased 3-stage OTA; 0.6 µm IGZO-on-plastic	Gain-boosted cross-coupled differential amplifier; 0.6 µm a-IGZO; flexible polyimide	Programmable-gain a-IGZO amplifier; flexible 27 µm polyimide	2-stage internally compensated op-amp; dual-gate self-aligned a-IGZO; flexible substrate	Positive-feedback op-amp; 5 µm a-IGZO; flexible substrate	All-enhancement op-amp; a-IGZO; 50 µm flexible polyimide
Supply Voltage	3.3 V	3.0 V	4.0 V	5.0 V	10 V	6.0 V	5.0 V
Power	33–53 μW	60 μW	320 μW	615.15 μW	2.43 mW	6.78 mW	900 μW
Gain	56.6 dB	41.2 dB	35.85 dB	3.20–20.09 dB	57 dB	19 dB	18.7 dB
Open-loop GBW	–	200 kHz	345 kHz	–	311 kHz	–	472 kHz
Closed-loop −3 dB bandwidth	3.7 kHz	147 kHz	–	1.16 MHz	–	25 kHz	–
Phase Margin	54.6° from peaking.	60.7°	65.4°	–	75°	70°	–
Load/Test	Unity-gain follower, 0.4 nF/setup)	CL = 40 pF. IB = 3 μA	10 MHz/3.9 pF probe	10 MΩ//8 pF	–	–	Bent to 5 mm radius
Area	0.128 mm^2^	0.042 mm^2^	0.075 mm^2^	–	3.69 mm^2^	25.2 mm^2^	–
FoM_S_ [MHz·pF/mA]	90–150	400	–	–	–	–	–

## Data Availability

Datasets used/analyzed in this study are available from the corresponding author on reasonable request.
